# Effect of the light and dark conditions on flower opening time between cultivated rice (*Oryza sativa*) and a near-isogenic early-morning flowering line

**DOI:** 10.1093/aobpla/plab040

**Published:** 2021-07-01

**Authors:** Tsutomu Ishimaru, Kazuhiro Sasaki, Ikuo Nozaki, Masanori Ichihashi, Hiroaki Shimizu, Masataka Wakayama, Hideyuki Hirabayashi

**Affiliations:** 1 Hokuriku Research Station, Central Region Agricultural Research Center, National Agriculture and Food Research Organization (CARC/NARO), Inada, Joetsu, Niigata 943-0193, Japan; 2 Japan International Research Center for Agricultural Sciences (JIRCAS), 1-1 Ohwashi, Tsukuba, Ibaraki 305-8686, Japan; 3 Institute for Advanced Biosciences, Keio University, 246-2 Mizukami, Kakuganji, Tsuruoka, Yamagata 997-0052, Japan; 4 NARO Institute of Crop Science, NARO, 2-1-18 Kannondai, Tsukuba, Ibaraki 305-8518, Japan

**Keywords:** Early-morning flowering, flower opening time, genetic control, light regulation, *Oryza sativa*, rice

## Abstract

Flower opening time (FOT) is affected by genetic and environmental factors, but little is known about the effect of light and dark conditions on FOT in cereal crops. FOT of an *indica* rice cultivar, IR64, and its near-isogenic line carrying a QTL for an early-morning flowering trait (IR64+*qEMF3*) were investigated in a natural-light and temperature-controlled small greenhouse by exposing either the panicle or stem or both plant organs to different light and dark conditions. FOT did not change in either genotype when panicles were exposed to light. A large difference in FOT was found between genotypes when panicles were exposed to dark conditions; no flower opening was observed in IR64, whereas flower opening was delayed but observed in IR64+*qEMF3*. These results suggest that the panicle is the organ that perceives light for flower opening in both genotypes, whereas the light requirement to reach flower opening was quite different between genotypes. Flower opening of IR64 occurred concomitantly with elongation of anther filament in the light after the dark treatment stopped, whereas approximately half of flowering of IR64+*qEMF3* resulted in apparent cleistogamy even during dark treatment. An extended duration of the dark treatment until 1730H (30–50 min before sunset) made FOT of IR64 spikelets on the next day shifted to a time as early as that of IR64+*qEMF3*, with significant advancement of FOT compared to the control IR64 spikelets. Our results indicated that different flowering responses to light and dark conditions exist between IR64 and IR64+*qEMF3*. These findings provide clues for understanding the unique genetic controls of flowering in an EMF line in rice. This study also showed evidence that artificial light environments can shift the FOT of IR64 to that of IR64+*qEMF3*.

## Teaser

Our study revealed that light and dark conditions significantly affect flower opening time in rice.

## Introduction

Flowering is the developmental stage most sensitive to heat stress in cereal crops. Heat-induced spikelet sterility is one of the biggest threats to crop production in the era of global warming (Prasad *et al*. 2017). Flower opening time (FOT) is very important in the context of a heat escape strategy; heat-induced spikelet sterility can be mitigated by shifting FOT to the cooler early-morning hours (Satake and Yoshida 1978; Ishimaru *et al*. 2010).

FOT is affected by both genetic and environmental factors in cereal crops. In the *Oryza* genus, wide genetic variation in FOT was found among wild species (Sheehy *et al*. 2005; Thanh *et al*. 2010). In cultivated rice, some accessions of *O. glaberrima* have an early-morning flowering (EMF) trait (Nishiyama and Blanco 1980). In contrast, very narrow genetic variation in FOT among cultivars of *O. sativa* has been reported; most cultivars of *O. sativa* flowered 3.5–5.5 hours after sunrise (HAS; Hirabayashi *et al*. 2015; Bheemanahalli *et al*. 2017). Environmental factors such as mean minimum air temperature, vapour pressure deficit and solar radiation in the early morning on the day of flowering are reported to have significant influence on FOT (Kobayasi *et al*. 2010; Julia and Dingkuhn 2012). High temperatures have also been reported to advance FOT in rice (Jagadish *et al*. 2008). In wheat, peak FOT shifts to the cooler hours of the day (i.e. early in the morning and late in the evening) under high temperature stress, thereby providing an alternative heat escape mechanism to the cultivars (Sun *et al*. 2018). However, little is known about the effect of light conditions on FOT in rice and wheat.

Several effects of light on FOT have been studied in *Pharbitis nil* (Japanese morning glory) in detail: a certain period of darkness is required (Kaihara and Takimoto 1979), interaction of light and temperature conditions critically affects FOT (Kaihara and Takimoto 1981a), and the petal midrib in the flower is the organ that perceives light and temperature for flower opening (Kaihara and Takimoto 1981b). It remains unclear whether these same parameters that influence FOT in Japanese morning glory are applicable to plants in the Poaceae family (e.g. rice).

Our group previously developed a near-isogenic line (NIL) carrying a QTL for an EMF trait (*qEMF3*) derived from a wild rice, *O. officinalis*, in the genetic background of the *indica* rice cultivar, IR64 (Hirabayashi *et al*. 2015). The developed NIL was designated as IR64+*qEMF3*. In the present study, we examined the effects of genetic factors, light conditions and their interaction on FOT in rice using IR64 that has a normal FOT and the NIL IR64+*qEMF3* that has a FOT of 1.0–2.0 earlier than IR64 (Hirabayashi *et al*. 2015; Bheemanahalli *et al*. 2017). The focus of this study was (i) to identify the organ(s) essential for perceiving light signal leading to flower opening, (ii) to characterize the different flowering responses to light and darkness between the genotypes that have contrasting FOTs and (iii) to determine the effect of dark treatment on the FOT of the next day. We hypothesized that different responses of flowering to light and dark conditions could be observed between IR64 and IR64+*qEMF3*. Unique mechanisms underlying the regulation of flowering in an EMF rice (IR64+*qEMF3*) is proposed.

## Materials and Methods

### Plant materials

**Figure 1.**
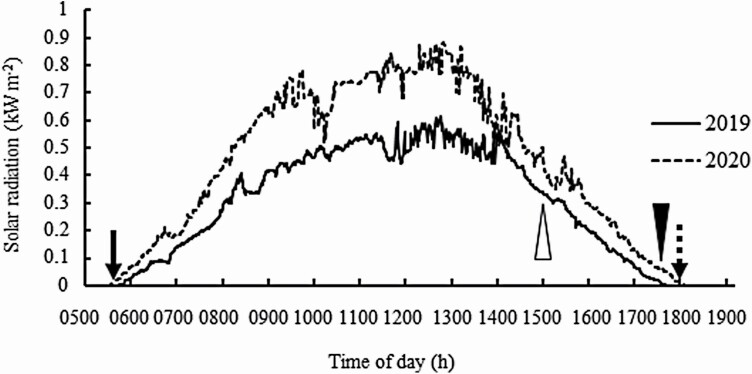
Solar radiation (kW m^−2^) during the flowering pattern observation in 2019 and 2020. Arrow and broken arrow indicate the approximate time of sunrise (0515–0530H) and sunset (1750–1820H), respectively. Empty arrowhead and black arrowhead indicate the time when dark treatment stopped at 1500H and 1730H in Experiments 2 and 3, respectively.

IR64, an *indica* rice cultivar, and IR64+*qEMF3*, a BC_3_-derived NIL in the IR64 genetic background (Hirabayashi *et al*. 2015), were used. Experiments were conducted at Hokuriku Research Station, Central Region Agricultural Research Center, National Agriculture and Food Research Organization (CARC/NARO, Joetsu, Niigata, Japan; 37′1°N, 138′3°E) in 2019 and 2020. Twelve seeds were sown uniformly in a circle in a 0.02-m^2^ pot following the method of Satake (1972). As a basal dressing, 1.5 g each of N–P_2_O_5_–K_2_O was applied per pot, and 1 g of ammonium sulfate per pot was applied as a top dressing as necessary. Plants were trimmed leaving only the main stems and were grown outdoors until the booting stage. At the booting stage, all pots were transferred into a small natural-light greenhouse that was air conditioned to provide a constant day–night temperature of 25 °C (4S-135-A, Koito Electric Industries, Ltd, Shizuoka, Japan). The temperature of the chamber was monitored every 10–15 min throughout each experiment by a thermo recorder (TR-52, T&D Corporation, Matsumoto, Japan). The average monitored temperature during the experiments was 23.9 °C ± SD = 1.3 and 24.2 °C ± SD = 1.4 in 2019 and 2020, respectively. Climatic factors other than temperature were not controlled inside the greenhouse. Solar radiation that was officially recorded every minute in the field of Hokuriku Research Station was averaged during the experiments in 2019 and 2020. The light level in the greenhouse was reduced by 11 % compared to the field light conditions due to the greenhouse glass. Sunrise and sunset during the experiments occurred at 0515–0530H and 1750–1820H, respectively (Japan Standard Time) (Fig. 1).

### Experiment 1: the flowering patterns of defoliated plants

**Figure 2.**
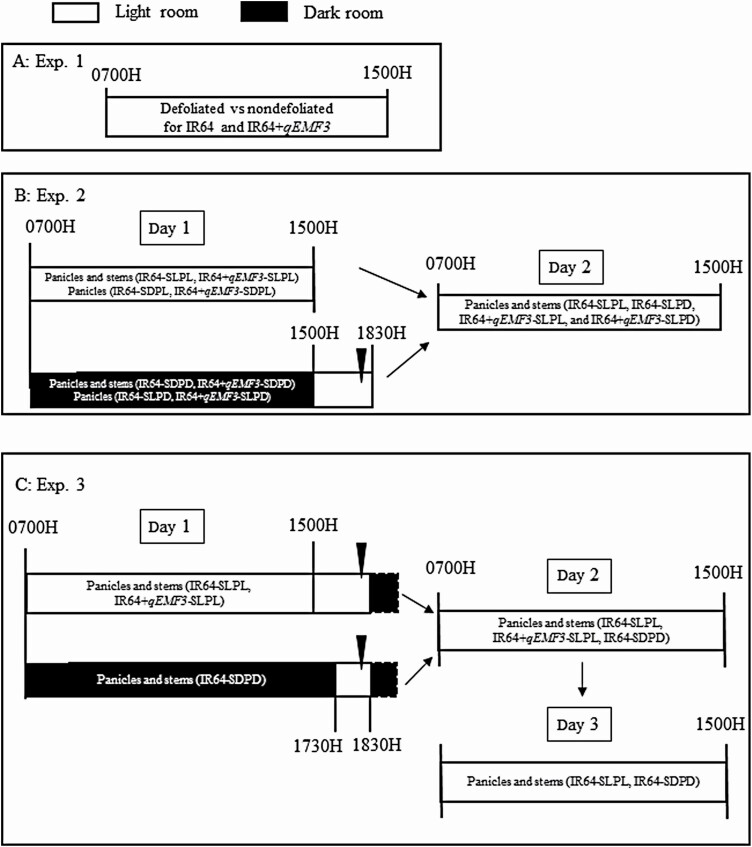
Schematic diagram of a sequence of flowering pattern observation for light and dark treatments in Experiments 1–3. Experiment 1 (A), Experiment 2 (B), and Experiment 3 (C). Empty bars indicate the time of light treatment, and black bars indicate the time of dark treatment. Black arrowheads in (B) and (C) indicate the approximate time of sunset (1800H). Black bars with a broken outline after 1830H on Day 1 indicate the natural dark condition. On Day 1 of Experiment 3, flowering pattern observation was conducted only using plants of IR64-PDLD.

At 1730–1800H (just before sunset) on the day prior to the experiment, all leaf blades were defoliated from the leaf sheaths and ligules [**see****Supporting Information—Fig. S1**]. Control plants were not defoliated. On the day scheduled for flowering pattern observations, opened spikelets on defoliated and nondefoliated plants were marked every 30 min with fine-tipped pens from 0700H to 1500H (Fig. 2A) based on the protocol reported by Hirabayashi *et al*. (2015). Three to four panicles that had exserted to approximately one-half of the entire panicle length from the leaf sheath were used for each genotype per day. One or two pots were used per genotype per day. The experiment was repeated 3 days for both genotypes using different pots every day. Nonexerted spikelets were not considered as a part of the observation.

### Experiment 2: the flowering patterns of panicles and stems individually exposed to light and dark conditions

**Figure 3.**
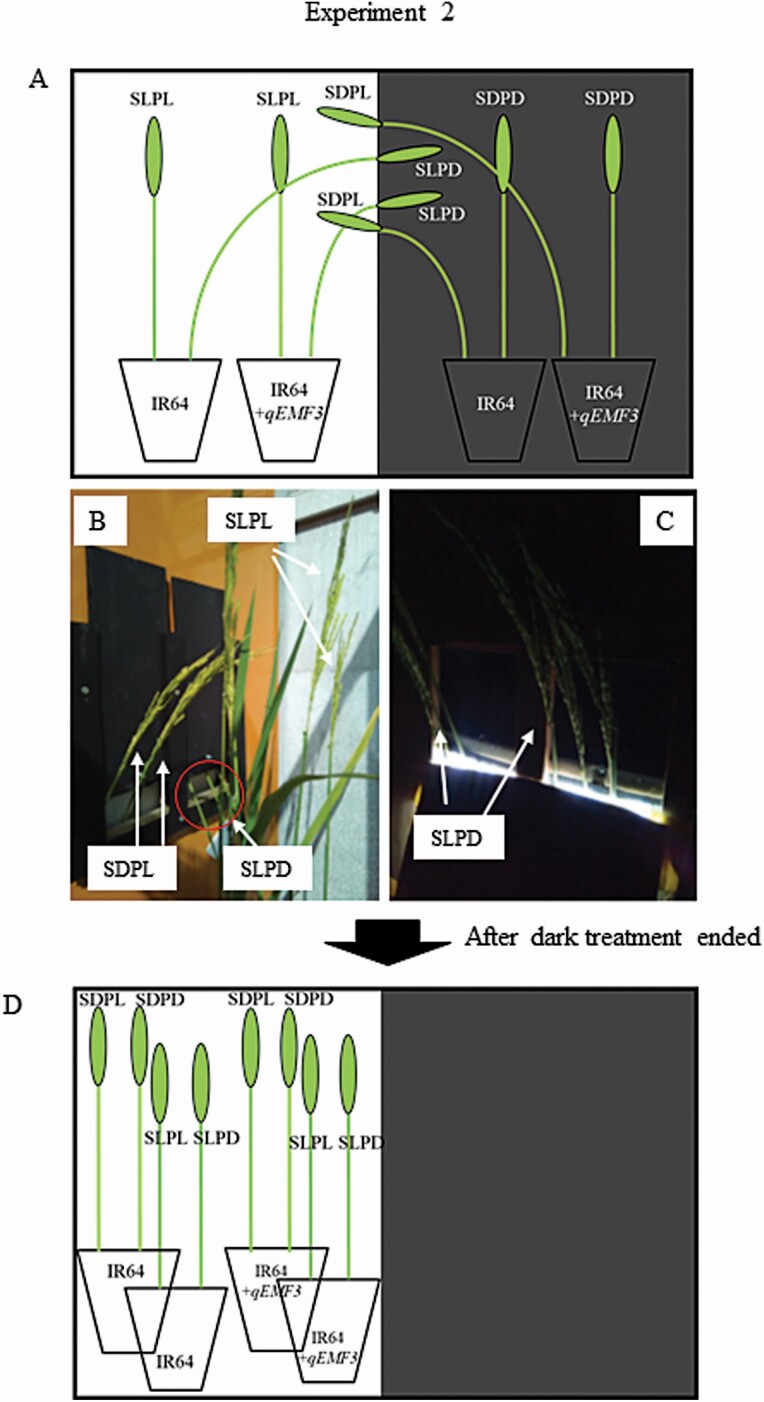
Schematic diagram showing how the exposure of stems and panicles to the light and dark conditions in a small greenhouse was achieved (A), and photographs of the light room (B) and dark room (C). Acronyms indicated near each panicle correspond to the treatments summarized in Table 1. Panicles of all treatments were subjected to light conditions after the dark treatment ended (D).

Flowering patterns were observed for two consecutive days (Day 1 and Day 2) (Fig. 2B). Defoliated plants prepared as described for Experiment 1 were used in this experiment. A natural-light greenhouse, set at a constant 25 °C, was divided into a light room and a dark room with a wooden panel with two slits at panicle height (Fig. 3A–C; **see****Supporting Information—Fig. S2**). Half of the greenhouse was covered with plastic film (Shiroshiro Coat 5, I-Agri Corp., Ibaraki, Japan; shading rate 99.9 % with a heat shielding effect) to create the dark room. A pre-dark room was constructed with a blackout curtain to prevent light from entering the dark room [**see****Supporting Information—Fig. S2**]. Based on our preliminary investigation, temperature in the light and dark rooms were 23.8 °C ± 0.4 and 23.7 °C ± 0.4, respectively (average of three time points ± SD). Difference in temperature between light and dark rooms was very low. Pots of both genotypes were placed in the light and dark rooms (Fig. 3A). Two or three panicles in pots from the light rooms were exposed to the dark through slits (stems remained in the light room), and two or three panicles in pots from the dark room were exposed to light through slits (stems remained in the dark room) (Fig. 3B and C). Thus, a total of eight treatments were established with different combinations of genotypes, light conditions and organs (Table 1; Fig. 3A). For instance, *IR64* plants with *S*tems in the *L*ight and *P*anicles in the *D*ark were designated as ‘IR64-SLPD’ (Table 1; Fig. 3A). Opened spikelets were marked every 30 min with fine-tipped pens from 0700H to 1500H in the light rooms (Fig. 2B). Visual inspection of flower opening in the dark room was feasible because of the very slight amount of light (2–4 μmol m^−2^ s^−1^ measured by a quantum flux meter, model MQ-200, Apogee Instruments, North Logan, UT) entering from the slits at panicle height (Fig. 3C). Once spikelets opened, it took more than 30 min until closure for some spikelets under the given temperature condition. To avoid double counting of already opened spikelets at the next 30-min observation in the dark, anthers that had dehisced from opened spikelets were carefully removed at every 30-min observation during the dark treatment. At 1500H, when the solar radiation was still high (Fig. 1), dark treatment ended, and all pots were transferred to the light treatment (Figs 2B and 3D). We found that dark room had elongated anther filaments without opening. In this study, such flowering style was defined as ‘apparent cleistogamy’. The number of apparent cleistogamous spikelets was counted on each panicle of IR64-SLPD, IR64+*qEMF3*-SLPD, IR64-SDPD and IR64+*qEMF3*-SDPD. Thereafter, opened spikelets were continuously monitored every 30 min until 1830H, when it became completely dark outside. Two to three panicles that had exserted to approximately one-half of the entire panicle length from the leaf sheath were used for each treatment per sequence. One or two pots were used per genotype per sequence.

**Table 1.** Acronyms for treatment based on genotypes and light conditions in Experiments 2 and 3.

Experiment	Genotype	Light condition		Acronyms for treatment	Remarks
		Stem	Panicle		
2	IR64	Light	Light	IR64-SLPL	Days 1 and 2: plants were placed in the light for flowering pattern observation.
	IR64+*qEMF3*			IR64+*qEMF3*-SLPL	
	IR64	Dark	Light	IR64-SDPL	Day 1: dark treatment ended at 1500H. Day 2: plants of IR64-SLPD and IR64+*qEMF3*-SLPD were used for flowering pattern observation.
	IR64+*qEMF3*			IR64+*qEMF3*-SDPL	
	IR64	Light	Dark	IR64-SLPD	
	IR64+*qEMF3*			IR64+*qEMF3*-SLPD	
	IR64	Dark	Dark	IR64-SDPD	
	IR64+*qEMF3*			IR64+*qEMF3*-SDPD	
3	IR64	Light	Light	IR64-SLPL	Days 1–3: plants were placed in the light.
	IR64+*qEMF3*			IR64+*qEMF3*-SLPL	
	IR64	Dark	Dark	IR64-SDPD	Day 1: plants were placed in the dark until 1730H.
		(Day 1)			
		Light	Light		Days 2 and 3: plants were placed in the light.
		(Days 2 and 3)			

On the next day (Day 2), plants in IR64-SLPD and IR64+*qEMF3*-SLPD on Day 1 were used to investigate their flowering pattern from 0700H to 1500H in the light (Fig. 2B). Flowering patterns of panicles that were used in IR64-SLPL and IR64+*qEMF3*-SLPL on Day 1 were also investigated as references for both genotypes (Fig. 2B). Nonexerted spikelets were not considered as a part of the observation. The same pots were used for a sequence of observation during Days 1 and 2, while different pots were used for every sequence of observations. The experiment was repeated three sequences per treatment. Note that the observations of the flowering pattern between Day 1 and Day 2 were made for IR64+*qEMF3* as an individual experiment in 2019 and 2020.

### Experiment 3: the flowering pattern of IR64 resulting from an extended period of dark treatment

Flowering patterns were observed for three consecutive days (Day 1, Day 2 and Day 3) per sequence (Fig. 2C). Defoliated plants prepared as described for Experiment 1 were used in this experiment. On Day 1, pots of IR64 were treated in the dark until 1730H (about 30–50 min before sunset) (Fig. 2C), thereby receiving an additional 2.5 h of dark treatment compared to the dark treatment in Experiment 2 (Fig. 2B). Solar radiation was already at the low level at 1730H (Fig. 1). Panicles for this experiment were designated as IR64-SDPD (Table 1). Observation for opened spikelets was conducted every 30 min in the dark room. When dark treatment ended at 1730H, pots were transferred to the light room, and the number of apparent cleistogamous spikelets was counted in each panicle. Thereafter, opened spikelets were continuously counted every 30 min until 1830H (Fig. 2C). After observation on Day 1 finished at 1830H, anthers whose spikelets opened by 1830H were removed. Flowering patterns were not observed from 1830H to 0630H, but the total number of spikelets that had opened during 1830H–0630H was counted at 0630H, before starting the observations of flowering patterns on Day 2. Spikelets whose anthers outside of the glumes were counted as opened spikelets during 1830H–0630H. Because the anthers of spikelets that had opened by 1830H on Day 1 were removed, spikelets that flowered during 1830H−0630H were easily distinguished. Note that plants of IR64-SLPL and IR64+*qEMF3*-SLPL were placed in the light throughout Day 1 to conduct the flowering pattern observation with those of IR64-SDPD on Day 2 (Fig. 2C).

On Day 2, opened spikelets of IR64-SDPD, IR64-SLPL and IR64+*qEMF3*-SLPL were marked every 30 min with fine-tipped pens from 0700H to 1500H in the light (Fig. 2C). On Day 3, the flowering patterns of IR64-SDPD and IR64-SLPL were investigated in the same method as Day 2 (Fig. 2C). One or two pots were used per treatment per sequence. Nonexerted spikelets were not considered as a part of the observation. The same pots were used for a sequence of observation during Days 1–3, while different pots were used for every sequence of observation. Sequence of observation was repeated three times for Days 1 and 2, and for two times for Day 3.

### Calculation of the time to reach 50 % of flower opening

Calculation of the time to reach 50 % of flower opening (FOT50) is based on the method of Hirabayashi *et al*. (2015). Briefly, FOT was represented in the ‘time (hour) after sunrise’ criterion considering flowering pattern observation on the different date. Based on the time-course graphs with cumulative flowering events, the time when 50 % of total observed flowering events occurred was calculated using fitted logistic curves estimated by nonlinear regression model using the R program (ver. 3.5.2). The exact time of sunrise was calculated using the Koyomi Station software (http://eco.mtk.nao.ac.jp/koyomi/index.html.en).

### Statistical analysis

The differences of means of FOT50 were analyzed using the *t*-test or Tukey test implemented in the Excel-tokei (ver. 3.21), across the sequential replicates of each experiment.

## Results

### Experiment 1: the effect of defoliation on FOT

**Figure 4.**
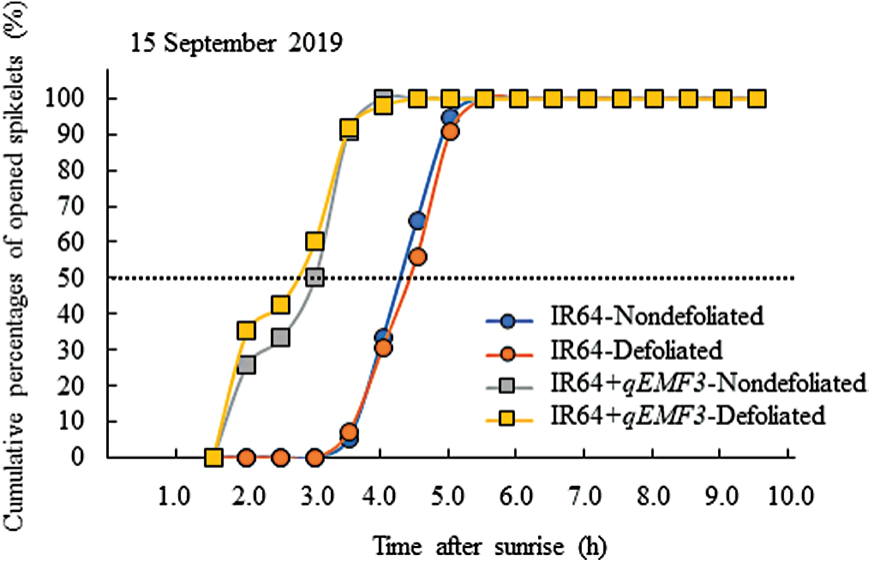
The representative flowering pattern of spikelets in defoliated and nondefoliated plants in Experiment 1. The observation date was 15 September 2019; % in each time point is calculated as (Spikelets that opened at a time point) ÷ (The sum of all spikelets that opened on that day) × 100. FOT50 of IR64 was 4.3 and 4.4 HAS for nondefoliated and defoliated plants, respectively, whereas FOT50 of IR64+*qEMF3* was 2.8 and 2.6 HAS for nondefoliated and defoliated plants, respectively, on that date.

Flowering patterns of defoliated and nondefoliated plants of IR64 and IR64+*qEMF3* were investigated (Fig. 4). On 15 September, flower opening of IR64 started after 3.0 HAS and finished by 5.5 HAS both in the defoliated and nondefoliated plants (Fig. 4). Flower opening of IR64+*qEMF3* started after 1.5 HAS and finished at about 4.0 HAS in both defoliated and nondefoliated plants (Fig. 4). Means of FOT50 were not different between the defoliated and nondefoliated plants in each genotype (Table 2).

**Table 2.** FOT50 of nondefoliated and defoliated plants in Experiment 1. Values are the mean ± SD of 3-day observation. ns, not significant by *t-*test.

Genotype	Treatment	FOT50	*t-*Test	Dates of observation
IR64	Nondefoliated	4.4 ± 0.5	ns	13–15 September 2019
	Defoliated	4.5 ± 0.6		
IR64+*qEMF3*	Nondefoliated	2.6 ± 0.7	ns	14–16 September 2019
	Defoliated	2.5 ± 0.7		

### Experiment 2: the effects of individual exposure of stems and panicles to light and dark conditions on FOT

**Figure 5.**
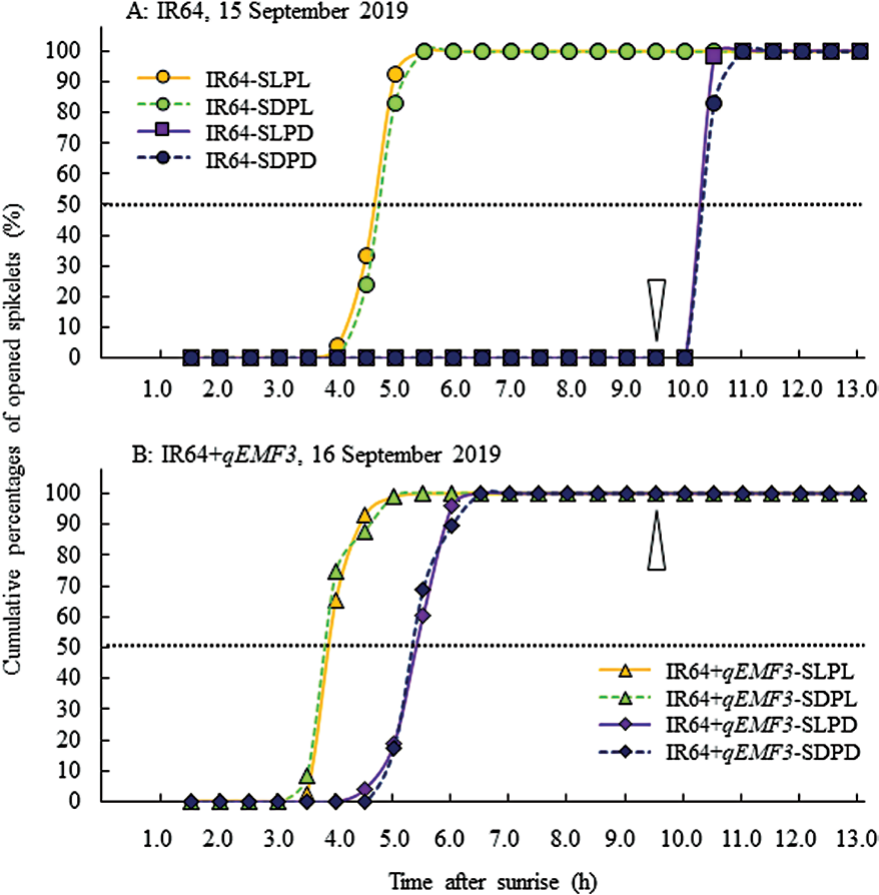
The representative flowering pattern of spikelets in treatments of Experiment 2. The observation date was 15 September 2019 for IR64 (A) and 16 September 2019 for IR64+*qEMF3* (B). Empty arrowheads indicate when the dark treatment ended for SLPD and SDPD in both genotypes; % in each time point is calculated as (Spikelets that opened at a time point) ÷ (The sum of all spikelets that opened on that day) × 100. FOT50 of IR64 was 4.6, 4.7, 10.3 and 10.3 HAS for SLPL, SDPL, SLPD and SDPD, respectively, on that date. FOT50 of IR64+*qEMF3* was 4.0, 3.9, 5.4 and 5.4 HAS for SLPL, SDPL, SLPD and SDPD, respectively, on that date.

**Figure 6.**
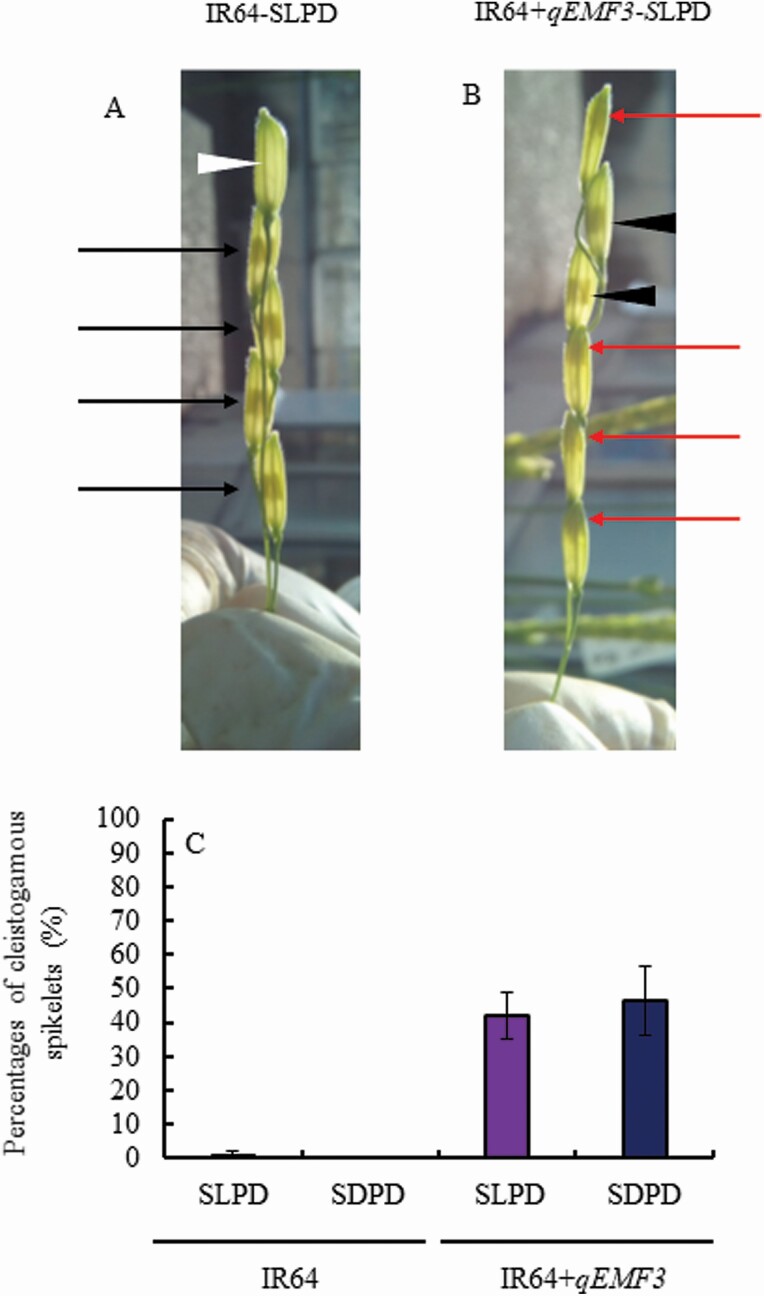
Photograph of spikelets on the primary rachis branches of IR64-SLPD (A) and IR64+*qEMF3*-SLPD (B), and the percentage of apparent cleistogamous spikelets in SLPD and SDPD in both genotypes (C) observed at 1500H. Black arrows in panel A indicate spikelets whose anthers remained at their original (middle) position inside the glumes prior to flowering at 1500H but opened around 1600–1700H thereafter. The white arrowhead in panel A indicates a spikelet that already flowered on the previous day. Red arrows in panel B indicate apparent cleistogamous spikelets, whereas in panel B, the black arrowheads indicate spikelets that did not flower on that day. In panel C, values are the mean ± SD (*n* = 3 days) of apparent cleistogamous spikelets in SLPD and SDPD in both genotypes; % of apparent cleistogamous spikelets is calculated as (Apparent cleistogamous spikelets) ÷ (The sum of opened and apparent cleistogamous spikelets) × 100. Photographs and data on the number of apparent cleistogamous spikelets were obtained at 1500–1515H.

Stems (culms, leaf sheaths and ligules) and panicles of IR64 and IR64+*qEMF3* were individually exposed to light and dark conditions (Fig. 3A–C), establishing a total of eight treatments in this experiment (Table 1). The flowering pattern of IR64-SLPL, whose panicles and stems of IR64 were exposed to light, was very similar to that of IR64-SDPL, in which only IR64 panicles were exposed to light; flower opening started after 4.0 HAS and finished by 5.5 HAS (Fig. 5A). In contrast, when panicles in IR64-SLPD and IR64-PDCD were exposed to dark conditions, the spikelets did not open until 10.0 HAS (Fig. 5A), and the anthers remained at the original (middle) position within spikelets (Fig. 6A). Apparent cleistogamous spikelets were rarely found in IR64-SLPD and IR64-SDPD (Fig. 6C). Spikelets of IR64-SLPD and IR64-SDPD simultaneously opened at 10.5 HAS, 1 h after the pots of these two treatments were transferred to light conditions at 9.5 HAS (Fig. 5A). As a consequence, means of FOT50 was significantly later in the treatments of SLPD and SDPD than those of SLPL and SDPL in IR64 (Table 3).

**Table 3.** FOT50 among treatments on Day 1 of Experiment 2. Values are the mean ± SD of 3-day observation. Different alphabets indicate the significant difference at the 5 % level by Tukey’s test. ns, not significant.

Genotype	Light condition	Day 1		
		FOT50	Tukey	Dates of observation
IR64	SLPL	5.5 ± 1.4	b	13–15 September 2019
	SDPL	5.7 ± 1.2	b	
	SLPD	10.4 ± 0.2	a	
	SDPD	10.5 ± 0.3	a	
IR64+*qEMF3*	SLPL	3.6 ± 0.4	ns	13, 15 and 16 September 2019
	SDPL	3.8 ± 0.1		
	SLPD	5.0 ± 0.5		
	SDPD	5.5 ± 1.0		

The flowering pattern of IR64+*qEMF3*-SLPL, in which IR64+*qEMF3* panicles and stems were exposed to light conditions, was very similar to that of IR64+*qEMF3*-SDPL, in which only IR64+*qEMF3* panicles were exposed to light conditions; flower opening started at 3.5 HAS and finished by 5.0 HAS (Fig. 5B). In spikelets of IR64+*qEMF3*-SLPD and IR64+*qEMF3*-SDPD, in which IR64+*qEMF3* panicles were exposed to darkness, flower opening started after 4.5 HAS until 6.5 HAS (Fig. 5B). As a consequence, means of FOT50 was later in the treatments of SLPD and SDPD than those of SLPL and SDPL in IR64+*qEMF3* although there was no significant difference between treatments (Table 3). Notably, 40–50 % of the spikelets in IR64+*qEMF3*-SLPD and IR64+*qEMF3*-SDPD of IR64+*qEMF3* showed apparent cleistogamy, in which the anthers extended to the top of spikelets when apparent cleistogamous spikelets were counted at 1500H (9.5 HAS) (Fig. 6B and C). Note that the time of day when apparent cleistogamy occurred in each spikelet could not be recorded because of the dark environment.

On the second day of Experiment 2 (Day 2), the flowering pattern of panicles in IR64-SLPD and IR64+*qEMF3*-SLPD, whose panicles were exposed to darkness until 1500H (9.5 HAS) on Day 1 (Fig. 2B), was investigated by comparing the flowering pattern of panicles in IR64-SLPL and IR64+*qEMF3*-SLPL, respectively. For IR64, the flowering pattern was very similar between IR64-SLPL and IR64-SLPD [**see****Supporting Information—Fig. S3A**] without significant difference in means of FOT50 [**see****Supporting Information—Table S1**]. For IR64+*qEMF3*, the flowering patterns was very similar between IR64+*qEMF3*-SLPL and IR64+*qEMF3*-SLPD [**see****Supporting Information—Fig. S3B**] without significant difference in means of FOT50 [**see****Supporting Information—Table S1**].

### Experiment 3: the effect of extending the dark treatment duration on the flowering pattern of IR64

Only 0–1.3 % of the spikelets in IR64-SDPD flowered before 1730H in the dark condition (Table 4). After the panicles were treated in the light (already low level of solar radiation at 0.03 and 0.06 kW m^−2^ in 2019 and 2020, respectively) starting from 1730H (~12.0 HAS) (Fig. 1), open spikelets were rarely observed on Day 1 (Table 4). During nighttime (1830–0630H on Day 1 and Day 2), 0–6.4 % of these spikelets opened (Table 4). No apparent cleistogamous spikelets were observed in IR64-SDPD. Thus, the percentage of opened spikelets from 0700H (Day 1) to 0630H (Day 2) was only 5.2 % averaged over 3 days of observations (Table 4).

**Table 4.** Percentage of opened spikelets from 0700H to 1730H (Day 1), 1730H to 1830H (Day 1), 1830H to 0630H (Day 1 and Day 2) and 0630–1500H (Day 2) of IR64-SDPD in Experiment 3. Percentage in each time period was calculated as (Spikelets that opened at a time period) ÷ (The sum of all spikelets that opened on Day 1 and Day 2) × 100. No apparent cleistogamous spikelets were observed.

Opened spikelets (%)	0700–1730H (Day 1)	1730–1830H (Day 1)	1830–0630H (Day 1 and Day 2)	0630–1500H (Day 2)
16 and 17 September 2019	0	0	6.4	93.6
31 August–1 September 2020	1.3	0	0	98.7
1 and 2 September 2020	0.9	0.9	6.2	92.0
Average	0.7	0.3	4.2	94.8

**Figure 7.**
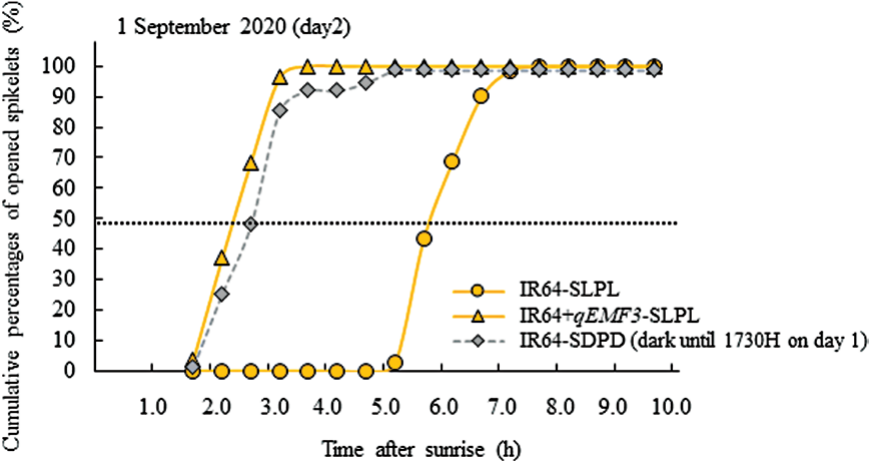
The representative flowering pattern of spikelets in IR64-SLPL, IR64+*qEMF3*-SLPL and IR64-SDPD on Day 2 in Experiment 3. The observation date was 1 September 2020; % in each time point is calculated as (Spikelets that opened at a time period) ÷ (The sum of all spikelets that opened on Day 1 and Day 2) × 100. FOT50 of IR64-SLPL, IR64+*qEMF3*-SLPL and IR64-SDPD was 5.9, 2.4, 2.7 HAS, respectively, on that date.

On Day 2 of 1 September 2020, spikelets in IR64-SDPD (dark until 1730H on Day 1) started flower opening after 1.5 HAS, and finished flower opening by 3.5 HAS, whereas spikelets in IR64-SLPL started flower opening after 5.0 HAS, and finished flower opening by 7.0 HAS in the light (Fig. 7). Spikelets in IR64+*qEMF3*-SLPL started flower opening after 1.5 HAS, and finished flower opening by 5.0 HAS in the light on that date (Fig. 7). Thus, the flowering patterns of spikelets in IR64-SDPD (dark until 1730H on Day 1) and spikelets in IR64+*qEMF3*-SLPL were similar without significant difference in means of FOT50, whereas those of spikelets in IR64-SLPL started flower opening much later than those of IR64-SDPD (dark until 1730H on Day 1) and IR64+*qEMF3*-SLPL on Day 2 with significant difference in means of FOT50 (Fig. 7; Table 5). On the third day (Day 3), the flowering pattern was similar between spikelets in IR64-SLPL and IR64-SDPD (dark until 1730H on Day 1 and light thereafter) in the light [**see****Supporting Information—Table S2** and **Fig. S4**].

**Table 5.** FOT50 among treatments on Day 2 of Experiment 3. Values are the mean ± SD of 3-day observation. Different alphabets indicate the significant difference at the 5 % level by Tukey’s test.

Genotype	Light condition	Day 2		
		FOT50	Tukey	Dates of observation
IR64	SLPL	5.5 ± 0.7	a	16 September 2019, and 1 and 2 September 2020
	SDPD	3.3 ± 0.7	b	
IR64+*qEMF3*	SLPL	3.0 ± 0.9	b	

## Discussion

### Panicles are the vital organ for perceiving light signal for flower opening

The aim of Experiment 1 was to determine whether rice leaf blades are involved in flower opening. Rice plants were defoliated just before sunset to eliminate any effects of light on the day prior to observation of the flowering patterns of defoliated and nondefoliated plants. Flowering patterns of nondefoliated and defoliated plants were very similar for IR64 and IR64+*qEMF3* (Fig. 4; Table 2), suggesting that leaf blades do not affect FOT. Defoliated plants in Experiment 1 had intact panicles and stems (culms, leaf sheaths and ligules) [**see****Supporting Information—Fig. S1**]. Therefore, we subsequently tested whether panicles and/or stems are the key organ(s) that determine FOT in rice plants in Experiment 2 by exposing both or either organ to light or dark conditions (Fig. 3A–C). FOT delayed to the later hours only when the panicles were exposed to dark in both genotypes as shown in the treatments of SLPD and SDPD in both IR64 and IR64+*qEMF3* (Fig. 5A and B; Table 3). The results obtained in Experiments 1 and 2 clearly indicate that panicles are the vital organs for perceiving light signal for flower opening even when the genotypes have contrasting FOTs. In *Pharbitis nil* (Japanese morning glory), cutting flower buds 1 day prior to flower opening of other buds does not change the FOT when compared with the FOT of intact flower buds on the plants, as long as the cut ends of the flower buds are immersed in water (Kaihara and Takimoto 1979). Flowers are the key organ for perceiving the necessary light signal for flower opening in rice and Japanese morning glories. In rice spikelets, flower opening physically occurs by rapid swelling of the lodicules (Hoshikawa 1993). In Japanese morning glories, petal midribs are the tissues that perceive the light (and also the temperature) for flower opening (Kaihara and Takimoto 1981b). Based on genetic studies of the Poaceae family, lodicules are hypothesized to be analogous to eudicot petals (see review by Yoshida and Nagato 2011). Lodicules are likely to be the key tissue for perceiving light required to open rice flowers. The physiological mechanism responsible for the interaction of light perception and rapid swelling of the lodicules needs to be investigated further in rice.

### Flower opening time of an *indica* rice cultivar, IR64, drastically changes by the light conditions

In IR64-SLPD and IR64-SDPD of Experiment 2, flower opening did not occur when panicles were exposed to the dark; flower opening occurred when panicles were treated with light for 1.0–1.5 h after being transferred to the light room (Fig. 5A). Even apparent cleistogamous spikelets were rarely observed in IR64-SLPD and IR64-SDPD (Fig. 6A and C). This result indicates that light is essential for flower opening in IR64. The observation that spikelets did not open when IR64 panicles were treated in the dark in Experiment 2 supports the hypothesis of a light requirement being necessary to achieve flower opening on the day of flowering as suggested by previous field studies (Kobayasi *et al*. 2010; Julia and Dingkuhn 2012). The flowering pattern on Day 2 of IR64-SLPD, whose panicles were exposed to darkness until 1500H on Day 1, clearly indicated that there was no effect of darkness on Day 1 influencing IR64 flower opening on Day 2 (**see****Supporting Information—Table S1** and**Fig. S3**]. This result may be because flower opening occurred simultaneously at 10.5 HAS in IR64-SLPD on Day 1 (Fig. 5A), and flower opening was completed for all spikelets that were expected to flower on that day.

In Experiment 3, the dark condition for IR64 was extended 2.5 h to 30–50 min before sunset (until 1730H) (Figs 1 and 2C). About 94.8 % of the spikelets opened on Day 2 in IR64-SDPD (dark until 1730H on Day 1) (Table 4). This result indicates that flower opening was not completed for most of the spikelets expected to flower on Day 1 and that light exposure to the panicles on Day 1 from 1730H to the time of natural darkness around 1830H did not reach the light requirement needed for IR64 flower opening. On Day 2, the flowering pattern of IR64-SDPD (dark until 1730H on Day 1) shifted significantly earlier than that of the control panicles in IR64-SLPL (Table 5; Fig. 7). This result suggests that light exposed to panicles on Day 1, even during a short period prior to sunset, might be effective in shifting the FOT to early morning with an additive effect of light exposure after sunrise on Day 2. The natural dark treatment after 1830H on Day 1 did not cancel the effect of light radiation received on Day 1; therefore, the flowering pattern in IR64-SDPD (dark until 1730H on Day 1) was markedly advanced on Day 2. In Japanese morning glories, flower opening starts 10 h after the onset of darkness at 23 °C or higher because the biological clock is regulated by the length of dark treatment (Kaihara and Takimoto 1981a). In the case of IR64, an *indica* rice cultivar, evidences showed that there is a carry-over of light signal perception over the night. The cumulative light radiation perceived by the panicle is hypothesized to be the trigger for flower opening. Surprisingly, the flowering pattern of spikelets in IR64-SDPD (dark until 1730H on Day 1) was very similar to the flowering pattern of spikelets of IR64+*qEMF3*-SLPL (Table 5; Fig. 7). The shift in flowering pattern to the early morning achieved by an artificial light–dark treatment (i.e. Experiment 3) and a genetic effect (i.e. *qEMF3*) is of particular interest for characterizing how FOT is controlled in rice. The detailed qualitative and quantitative investigation about the interactive effect of the light and dark treatments on the change of FOT in IR64 also needs to be further conducted.

The flowering pattern in the spikelets of IR64-SDPD (dark until 1730H on Day 1 and light thereafter) on Day 3 was similar to that in the spikelets of IR64-SLPL in Experiment 3 [**see****Supporting Information—Table S2** and **Fig. S4**]. This result together with the flowering pattern result on Day 2 of Experiment 2 [**see****Supporting Information—Table S1** and **Fig. S3**] indicate that light radiation in the early morning hours on the day of flowering critically affected the FOT for both genotypes.

### Flowering observation in darkness elucidates the unique mechanism regulating flowering in IR64+*qEMF3*

In Experiment 2, a clear genetic difference in the light requirement for flower opening was observed between IR64 and IR64+*qEMF3* in darkness; spikelets did not open in IR64-SLPD and IR64-SDPD, whereas approximately one-half of the spikelets opened in IR64+*qEMF3*-SLPD and IR64+*qEMF3*-SDPD (Figs 5 and 6). The flowering pattern of spikelets in IR64+*qEMF3*-SLPD and IR64+*qEMF3*-SDPD, whose panicles were treated in the dark, was slightly delayed compared to that of IR64+*qEMF3* spikelets of IR64+*qEMF3*-SLPL and IR64+*qEMF3*-SDPL, whose panicles received light (Table 3; Fig. 5B). This result suggests that darkness also affected the FOT in IR64+*qEMF3* to some extent. Very interestingly, approximately one-half of the remaining IR64+*qEMF3* spikelets in IR64+*qEMF3*-SLPD and IR64+*qEMF3*-SDPD showed apparent cleistogamy (Fig. 6B and C), a condition that was rarely observed in IR64 in any of the three experiments. These results provide us two critical insights about the unique mechanism of flowering in IR64+*qEMF3* in response to the light and dark treatments. First, a very low light requirement is needed to accomplish flower opening in IR64+*qEMF3*, and second, the flower opening and anther filament elongation are controlled in the different processes in IR64+*qEMF3*.

A wild rice, *O. officinalis* (IRGC Acc. 100947), is the donor of the EMF trait (Ishimaru *et al*. 2010). This accession of *O. officinalis* starts flower opening 1.2 h after the sunrise under natural summer conditions in Japan (Ishimaru *et al*. 2010). A QTL for EMF (*qEMF3*) was detected and transferred to an *indica* rice cultivar, IR64 (Hirabayashi *et al*. 2015). In nature, accessions of *O. officinalis* tend to inhabit shaded areas such as shallow ponds in the forest (Vaughan 1994). The very low light requirement to reach flower opening in IR64+*qEMF3* may be inherited from the preferred shade habitat of *O. officinalis* through the *qEMF3* locus.

In darkness, approximately one-half of the spikelets in IR64+*qEMF3*-SLPD and IR64+*qEMF3*-SDPD showed apparent cleistogamy, whereas apparent cleistogamous spikelets were very rare in IR64-SLPD and IR64-SDPD (Fig. 6A–C). In cultivated rice, the rapid swelling of lodicules and the anther filament elongation synchronously occurs due to the drastic inflow of water to these organs (Hoshikawa 1993). In IR64+*qEMF3*-SLPD and IR64+*qEMF3*-SDPD, these two biological processes, however, asynchronously occurred in the dark. Since the apparent cleistogamous spikelets were specifically observed in IR64+*qEMF3* in the dark, IR64+*qEMF3* appeared to allocate water to the male-associated organs such as anthers and filaments rather than the lodicules at flowering in the dark. So far, phenotyping of the EMF trait has involved monitoring the spikelets that reached flower opening (Ishimaru *et al*. 2010, 2012; Hirabayashi *et al*. 2015; Bheemanahalli *et al*. 2017). Flower opening itself is, however, just a consequence of lodicule swelling. The apparent cleistogamous spikelets of IR64+*qEMF3* under dark conditions provide a unique regulation of decoupling of flower opening and anther filament elongation in an EMF rice.

## Conclusions

This study explored the genetic and environmental (light) effects on FOT in rice. Our focus was (i) to identify the organ(s) critical for light signal perception leading to flower opening, (ii) to understand the differences in flowering response to light and dark conditions between genotypes having contrasting FOTs and (iii) to determine the effect of dark treatment on the FOT on the next day. Regarding goal (i), we found that the panicle is the organ that perceives light signal for flower opening in both genotypes. Regarding goal (ii), exposing panicles to darkness revealed quite different flowering responses between genotypes; IR64 spikelets opened 1.0–2.0 h after ending the dark treatment at 1500H, whereas one-half of the IR64+*qEMF3* spikelets opened even in the darkness. Notably, the remaining one-half of spikelets of IR64+*qEMF3* exhibited apparent cleistogamy. Together with findings from (i) and (ii), we determined that panicle is the organ to make a difference in FOT between IR64 and IR64+*qEMF3*, but different flowering responses to light and dark conditions exist between genotypes. An EMF trait attributed by the single QTL, *qEMF3*, may be determined by the function in the spikelets. Regarding goal (iii), FOT did not change when the dark treatment ended at 1500H on the day prior to observation. An extension of the dark treatment to 1730H, just 30–50 min before sunset, however, did not open the majority of IR64 spikelets. The following day, the FOT of the IR64 spikelets (IR64-SDPD) was significantly earlier than that of the control, IR64 spikelets that had been exposed to continuous light on the previous day (IR64-SLPL). Result from (iii) is potentially relevant for seed production, especially for hybrid cultivars, through environmental control of FOT. Overall, our results suggest a unique means to genetically control flowering in an EMF line of rice and environmental control of FOT in IR64 by imposing an artificial dark treatment.

## Supporting Information

The following additional information is available in the online version of this article—

**Table S1.** FOT50 among treatments in IR64 and IR64+*qEMF3* on Day 2 of Experiment 2.

**Table S2.** FOT50 among treatments on Day 3 of Experiment 3.

**Figure S1.** A photograph of the defoliated plants.

**Figure S2.** Design of the light and dark rooms in a greenhouse.

**Figure S3.** The representative flowering patterns of spikelets in SLPL and SLPD for IR64 (A, 15 September 2019) and SLPL and SLPD for IR64+*qEMF3* (B, 27 August 2020) on Day 2 of Experiment 2.

**Figure S4.** The representative flowering pattern of spikelets in IR64-SLPL and IR64-SDPD (dark until 1730H on Day 1 and light thereafter) on Day 3 of Experiment 3..

plab040_suppl_Supplementary_Materials_S1

plab040_suppl_Supplementary_Materials_S2

## Data Availability

Authors confirm that the data supporting the findings of this study are available as Supporting Information.

## Notes

**Phenome, Genome & Environment. Chief Editor:** Colleen Doherty
