# Supplementary material for: Effect of the light and dark conditions on flower opening time between cultivated rice (Oryza sativa) and a near-isogenic early-morning flowering line
Source: AoB Plants. 2021 Jul 1;13(4):plab040. doi: 10.1093/aobpla/plab040 (PMC8300546; doi:10.1093/aobpla/plab040)
Supplement: plab040_suppl_Supplementary_Materials_S1 [file plab040_suppl_supplementary_materials_s1.docx]

Supplementary Table S1 FOT50 among treatments in IR64 and IR64+*qEMF3* on Day 2 of Experiment 2.

| Genotype | Light condition | Day 2 | | |
| --- | --- | --- | --- | --- |
|  |  | FOT50 | *t-*test | Dates of observation |
| IR64 | SLPL | 4.8 ± 0.8 | ns | 15, 16 September 2019, and 5 September 2020 |
|  | SDPL | - |  |  |
|  | SLPD | 5.0 ± 0.7 |  |  |
|  | SDPD | - |  |  |
|  |  |  |  |  |
| IR64+*qEMF3* | SLPL | 3.6 ± 0.1 | ns | 26, 27, 29 August 2020 |
|  | SDPL | - |  |  |
|  | SLPD | 3.4 ± 0.1 |  |  |
|  | SDPD | - |  |  |

Values are the mean ± SD of three days observation.

ns; not significant by *t-*test.

Supplementary Table S2 FOT50 among treatments on Day 3 of Experiment 3.

| Genotype | Light condition | Day 3 | |
| --- | --- | --- | --- |
|  |  | FOT50 | Dates of observation |
| IR64 | SLPL | 4.3 | 2 and 3 September 2020 |
|  | SDPD | 4.6 |  |
| IR64+*qEMF3* | SLPL | - |  |

Values are the mean of two days observation.
