# Supplementary material for: Effect of the light and dark conditions on flower opening time between cultivated rice (Oryza sativa) and a near-isogenic early-morning flowering line
Source: AoB Plants. 2021 Jul 1;13(4):plab040. doi: 10.1093/aobpla/plab040 (PMC8300546; doi:10.1093/aobpla/plab040)
Supplement: plab040_suppl_Supplementary_Materials_S2 [file plab040_suppl_supplementary_materials_s2.pptx]

## Slide 1
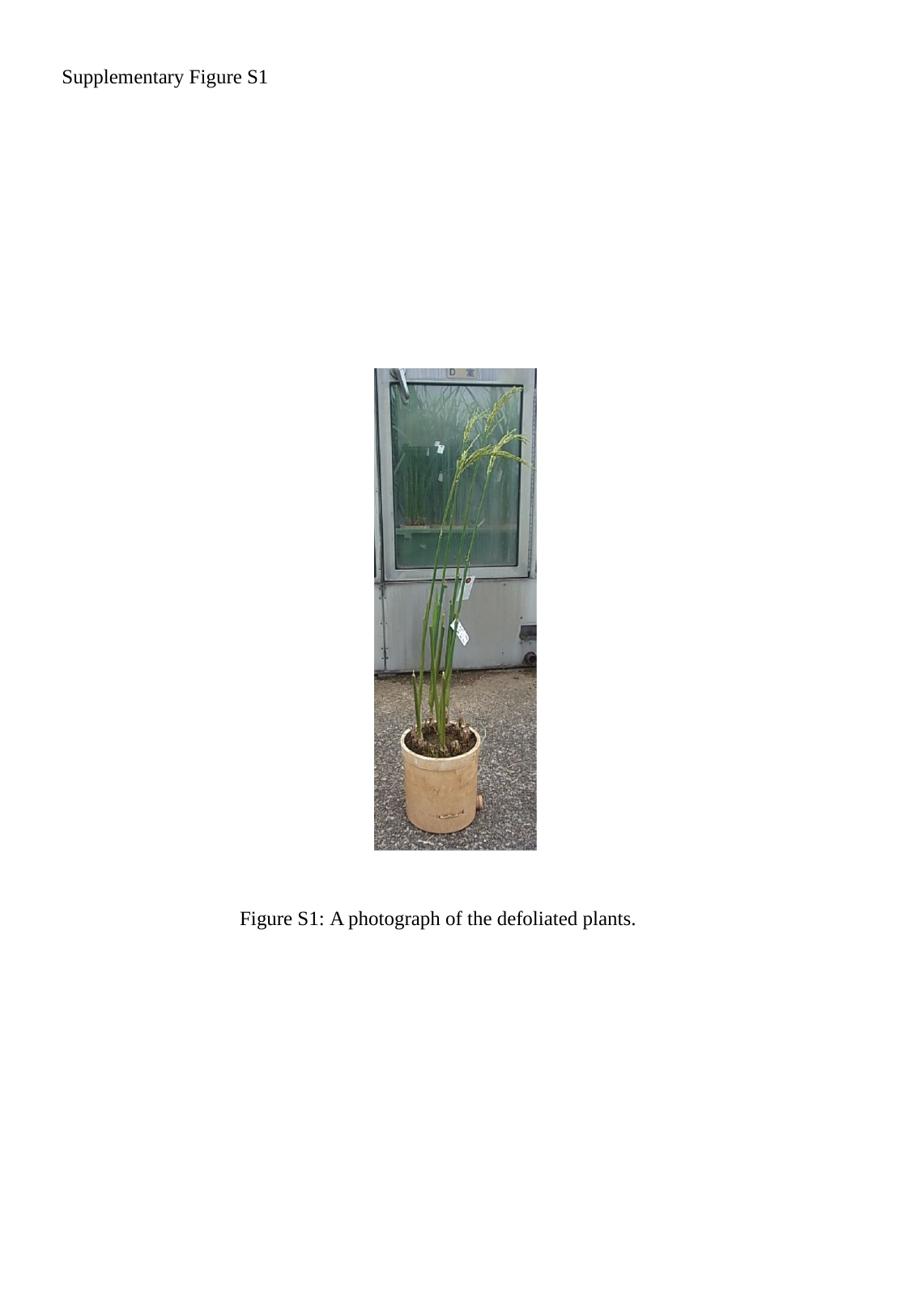

Supplementary Figure S1
Figure S1: A photograph of the defoliated plants.

## Slide 2
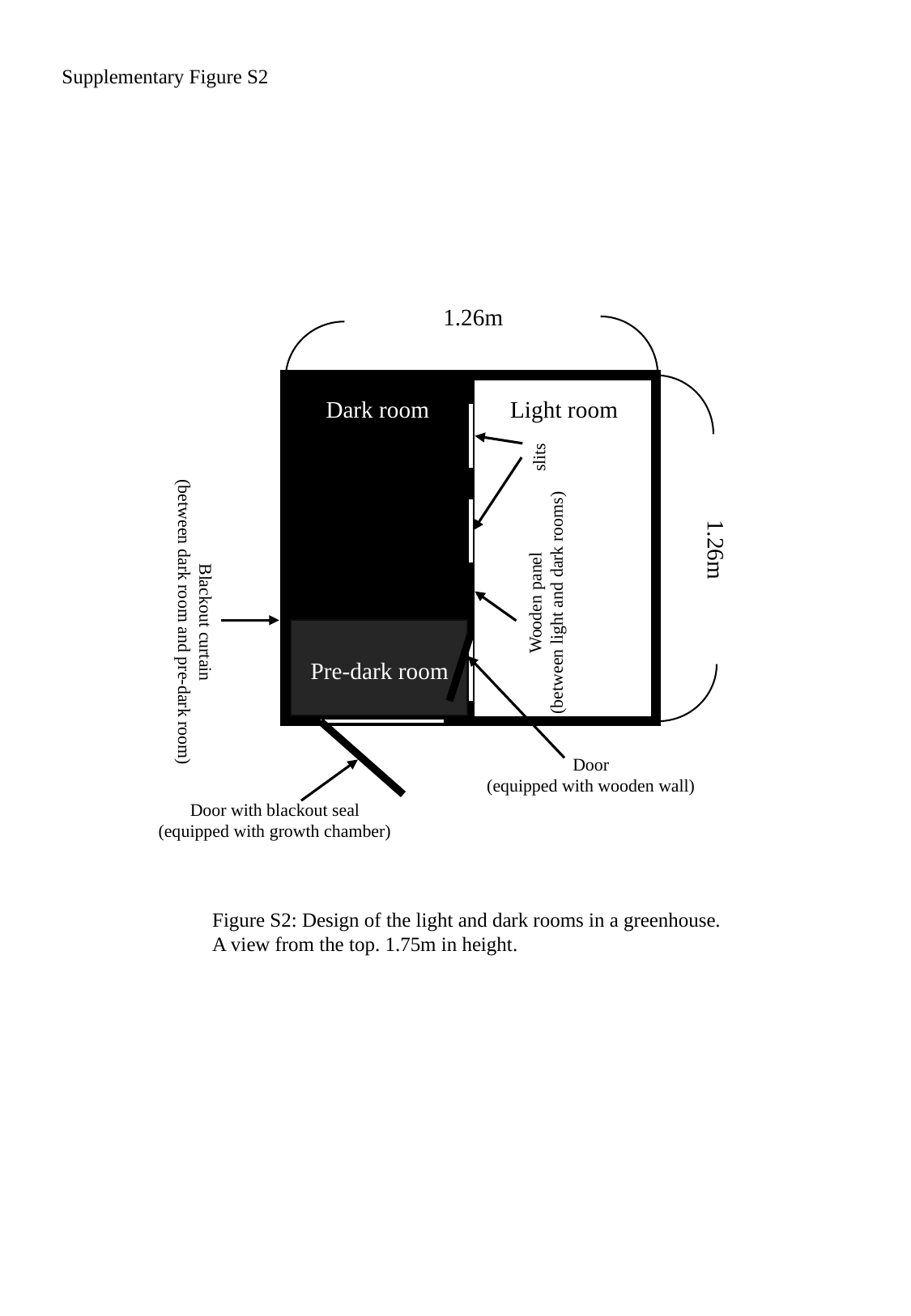

Supplementary Figure S2
1.26m
Dark room
Light room
slits
1.26m
Wooden panel
(between light and dark rooms)
Blackout curtain
(between dark room and pre-dark room)
Pre-dark room
Door
(equipped with wooden wall)
Door with blackout seal
(equipped with growth chamber)
Figure S2: Design of the light and dark rooms in a greenhouse. A view from the top. 1.75m in height.

## Slide 3
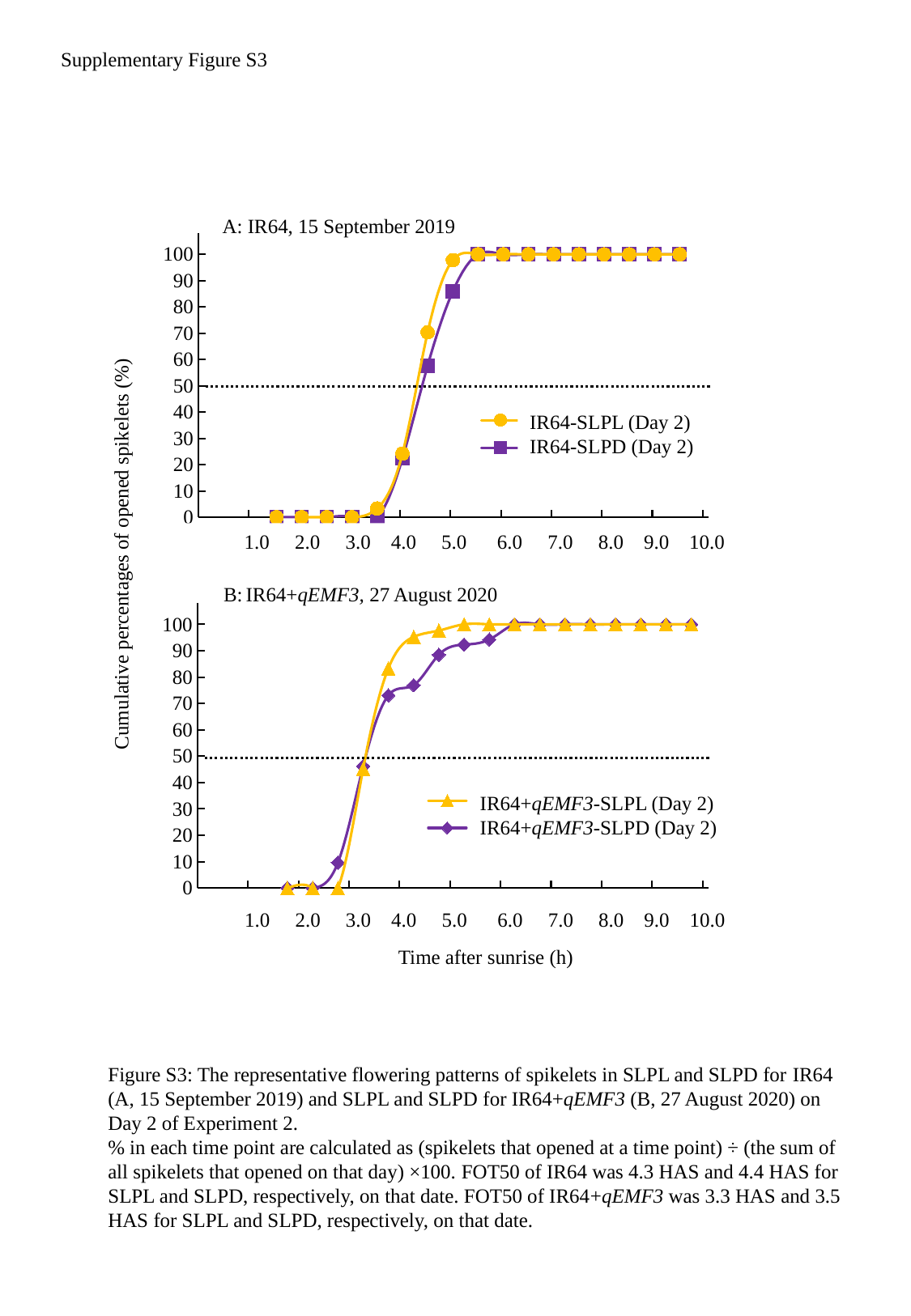

Supplementary Figure S3
A: IR64, 15 September 2019
### Chart
| Category | IR64-9/14-明 | IR64-9/14-暗 |
|---|---|---|IR64-SLPL (Day 2)
IR64-SLPD (Day 2)
 1.0 2.0 3.0 4.0 5.0 6.0 7.0 8.0 9.0 10.0
Cumulative percentages of opened spikelets (%)
B: IR64+qEMF3, 27 August 2020
### Chart
| Category | EMF-8/26-明 | EMF-8/26-暗 |
|---|---|---|IR64+qEMF3-SLPL (Day 2)
IR64+qEMF3-SLPD (Day 2)
 1.0 2.0 3.0 4.0 5.0 6.0 7.0 8.0 9.0 10.0
Time after sunrise (h)
Figure S3: The representative flowering patterns of spikelets in SLPL and SLPD for IR64 (A, 15 September 2019) and SLPL and SLPD for IR64+qEMF3 (B, 27 August 2020) on Day 2 of Experiment 2.
% in each time point are calculated as (spikelets that opened at a time point) ÷ (the sum of all spikelets that opened on that day) ×100. FOT50 of IR64 was 4.3 HAS and 4.4 HAS for SLPL and SLPD, respectively, on that date. FOT50 of IR64+qEMF3 was 3.3 HAS and 3.5 HAS for SLPL and SLPD, respectively, on that date.

## Slide 4
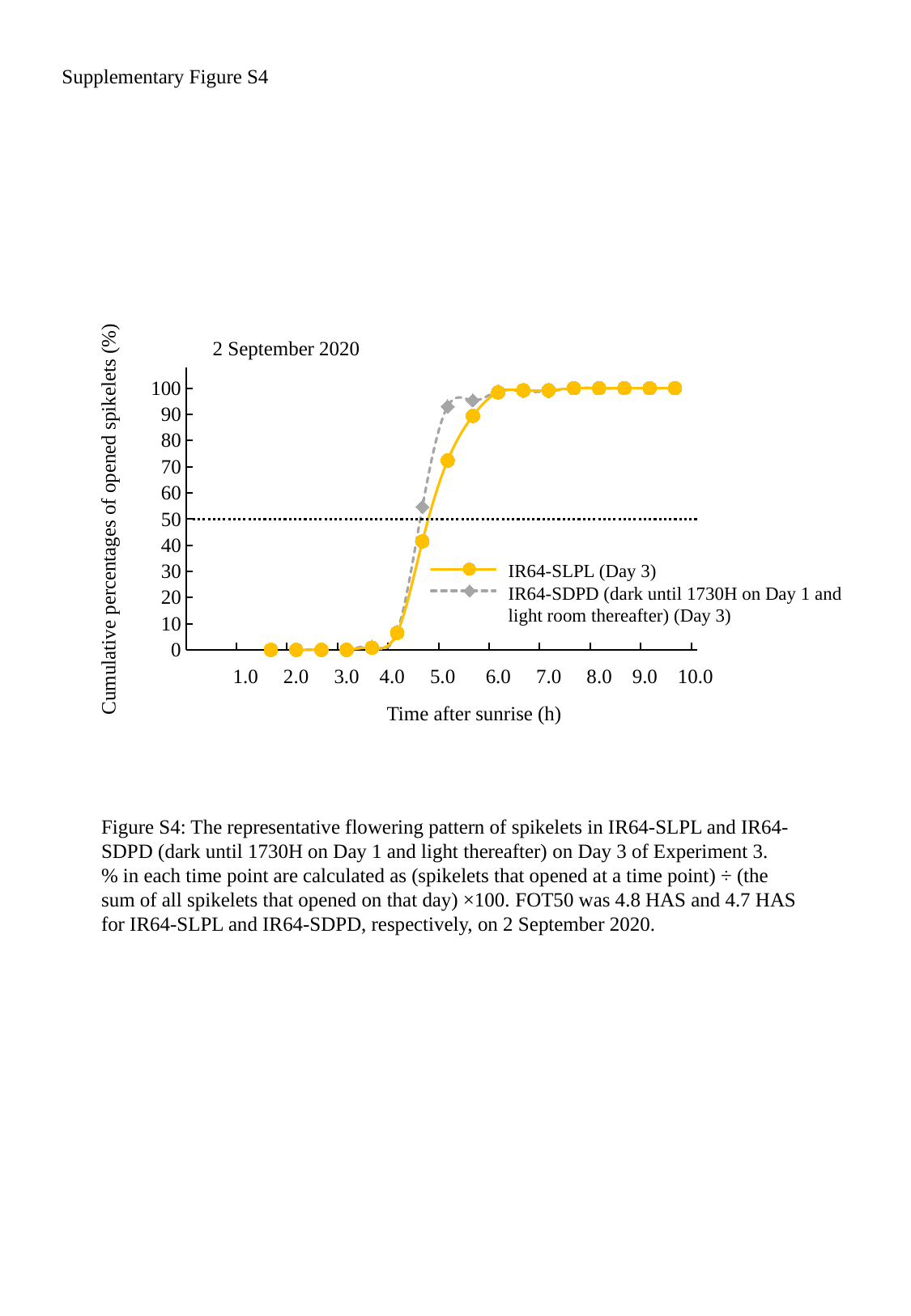

Supplementary Figure S4
2 September 2020
### Chart
| Category | IR64-Con | IR64-1730H-明-明 |
|---|---|---|Cumulative percentages of opened spikelets (%)
IR64-SLPL (Day 3)
IR64-SDPD (dark until 1730H on Day 1 and
light room thereafter) (Day 3)
 1.0 2.0 3.0 4.0 5.0 6.0 7.0 8.0 9.0 10.0
Time after sunrise (h)
Figure S4: The representative flowering pattern of spikelets in IR64-SLPL and IR64-SDPD (dark until 1730H on Day 1 and light thereafter) on Day 3 of Experiment 3.
% in each time point are calculated as (spikelets that opened at a time point) ÷ (the sum of all spikelets that opened on that day) ×100. FOT50 was 4.8 HAS and 4.7 HAS for IR64-SLPL and IR64-SDPD, respectively, on 2 September 2020.
